# Longitudinal functional connectivity changes across the clinical spectrum of *C9orf72* hexanucleotide repeat expansion carriers

**DOI:** 10.1002/alz70856_105253

**Published:** 2026-01-09

**Authors:** Liwen Zhang, Suvi Häkkinen, Youjin Jung, Maria Luisa Mandelli, Dana Leichter, Chiadi U. Onyike, Julio C. Rojas, Maria Luisa Gorno Tempini, Jennifer S. Yokoyama, Brad F. Boeve, Adam L. Boxer, Leah K. Forsberg, Hilary W. Heuer, Kejal Kantarci, Eliana Marisa Ramos, Howard J. Rosen, Bruce L. Miller, William W. Seeley, Taru M. Flagan, Suzee E. Lee, ARTFL/LEEFTDS/ALLFTD Consortia

**Affiliations:** ^1^ Institute for Medical Imaging Technology, Ruijin Hospital, Shanghai Jiao Tong University School of Medicine, Shanghai, China; ^2^ Memory and Aging Center, Department of Neurology, Weill Institute for Neurosciences, University of California, San Francisco, San Francisco, CA, USA; ^3^ Johns Hopkins University School of Medicine, Baltimore, MD, USA; ^4^ Department of Neurology, Memory and Aging Center, University of California San Francisco, San Francisco, CA, USA; ^5^ Mayo Clinic, Rochester, MN, USA; ^6^ Memory and Aging Center, Weill Institute for Neurosciences, University of California, San Francisco, San Francisco, CA, USA; ^7^ David Geffen School of Medicine, University of California, Los Angeles, Los Angeles, CA, USA

## Abstract

**Background:**

A hexanucleotide repeat expansion in *C9orf72* is the leading genetic cause of frontotemporal lobar degeneration and amyotrophic lateral sclerosis. There remains a critical need for biomarkers that track disease progression across clinical stages. Cross‐sectionally, intrinsic connectivity networks (ICNs) derived from task‐free fMRI (tf‐fMRI) detects abnormalities in *C9orf72* expansion carriers (*C9orf72+*), even when brain structural differences are subtle (Lee *et al.*, 2017). Few studies have examined longitudinal connectivity changes in *C9orf72+*, however.

**Methods:**

We analyzed longitudinal structural and tf‐fMRI data and plasma neurofilament light chain (NfL) concentrations of *C9orf72+* recruited from UCSF and the ALLFTD Consortia. Mean time from first to last scan was 2.4±1.3 years. Participants included 36 asymptomatic (aSx‐*C9*), 17 prodromal (prodromal‐*C9*), 29 symptomatic (Sx‐*C9*) carriers, and 107 healthy controls (HC). For structural MRI analyses, smoothed gray matter maps were parcellated into 246 regions‐of‐interest based on the Brainnetome atlas. We conducted voxelwise seed‐based tf‐fMRI analyses to examine ICNs showing alterations *C9orf72+* based on previous literature: salience network (SN), sensorimotor network (SMN), default mode network (DMN), and a medial pulvinar thalamus network (pulvinar). General linear models and linear mixed models (LMM) compared *C9orf72+* groups to HC in baseline and longitudinal gray matter and functional connectivity, as well as examined associations between longitudinal functional connectivity changes and baseline NfL concentrations.

**Results:**

Despite a lack of longitudinal gray matter decline, aSx‐ and Sx‐*C9orf72+* showed functional connectivity changes. Compared to HC, aSx‐*C9* had baseline hypoconnectivity in the SN, SMN, and pulvinar network. Longitudinally, this group showed declines in the SN and pulvinar network, alongside DMN increases. Prodromal‐*C9* exhibited baseline SMN hypoconnectivity and hyperconnectivity in the DMN and pulvinar network, but no significant longitudinal changes. Sx‐*C9* showed baseline hypoconnectivity in SN, SMN, and DMN, with additional regions of DMN hyperconnectivity, while longitudinally, SN and SMN connectivity increased. In aSx‐*C9*, higher baseline NfL concentrations correlated with regions of SMN decreases over time and both increases and decreases in the pulvinar network. In prodromal‐*C9* and Sx‐*C9* combined, higher NfL was associated with DMN increases longitudinally.

**Conclusions:**

Though gray matter declines were undetectable over a span of several years, functional connectivity networks changed dynamically in *C9orf72+*.

